# Current and future perspective on antimicrobial and anti-parasitic activities of *Ganoderma* sp.: an update

**DOI:** 10.1080/21501203.2017.1324529

**Published:** 2017-05-24

**Authors:** Buddha Bahadur Basnet, Li Liu, Li Bao, Hongwei Liu

**Affiliations:** ^a^ State Key Laboratory of Mycology, Institute of Microbiology, Chinese Academy of Sciences, Beijing, People’s Republic of China; ^b^ International College, University of Chinese Academy of Sciences, Beijing, People’s Republic of China; ^c^ Savaid Medical School, University of Chinese Academy of Sciences, Beijing, People’s Republic of China

**Keywords:** *Ganoderma* sp, antimicrobial, anti-parasitic, triterpenoid, quinone structures

## Abstract

Medicinal mushroom *Ganoderma* sp. is considered to be a key source for the production of therapeutic agents. Our current review indicates that a limited number (<19%; 79 out of >430) of isolated compounds have been tested and known to be active against several microorganisms and parasites. In this review, we aim to summarise all the antimicrobial and anti-parasitic works on *Ganoderma* sp. displayed on web of science, google scholar and endnote X7 from 1932 to August 2016. We further present and discuss the structure of active compounds against microorganisms and parasites. In addition, we also discuss the possible further research to identify lead compounds from *Ganoderma* sp. as a novel strategy to combat the potential global emergence of bad bugs and parasites.

## 1. Introduction

*Ganoderma* sp. is a medicinal mushroom producing a group of frequently studied bioactive compounds. They belong to the kingdom of Fungi, division of Basidiomycota, class of Agaricomycetes, order Polyporales, family of Ganodermataceae and genus of Ganoderma. A search for “Ganoderma” in the database Index Fungorum displayed 409 species records, including synonyms (http://www.speciesfungorum.org). *Ganoderma* sp., especially *G. lucidum, G. tsugae* and *G. applanatum,* are well studied and have been in use in East Asian countries since the ancient times for the treatment of various diseases (Ofodile et al. 2005; Paterson 2006; Ferreira et al. 2015). Triterpenes and polysaccharides are considered key constituents isolated from fruiting bodies, gills, spores and mycelia for their bioactivities (Xia et al. 2014).

Literature reviews suggest, besides its antimicrobial activities, *Ganoderma* sp. components exhibit a variety of bioactivities, including anti-tumour, immune-modulatory, antioxidant, antihypertensive and anti-androgenic. Moreover, *Ganoderma* sp. is widely used for the remedy of various chronic diseases such as cancers, diabetes, hypertension and hepatitis (Ofodile et al. 2005; Zhang et al. 2015). To date, most of the reviews on *Ganoderma* sp. have been focused on its anticancer and antioxidant activities and immune modulation (Sanodiya et al. 2009). Therefore, our basic aim is to provide a glimpse on the antimicrobial and anti-parasitic activities of *Ganoderma* sp. In addition, we also provide possible future prospect for research on *Ganoderma* sp. and its compounds.

In this review, we have performed literature searches in English (ISI Web of Science and Google Scholar) and Endnote X7 (online search, Pub Med) to find publications that described *Ganoderma* sp. for antimicrobial activities. We have used the keywords “Ganoderma” and “Antimicrobial”. Finally, we filtered individual references to determine the relevancy to our study. The inclusion criterion was the study that provided data or results or discussion on the antimicrobial activities of *Ganoderma* sp.

## 2. Antimicrobial and anti-parasitic bioactive compounds

*Ganoderma* sp. has been reported as important sources of antimicrobial bioactive compounds. Terpenes, terpenoids and polyketides of farnesyl quonines types are the major secondary metabolites (SMs) produced by *Ganoderma* sp. In *Ganoderma* sp., more than 316 terpenes have been reported, with the majority of compounds from *G. lucidium* (Xia et al. 2014).

**Figure 1.**
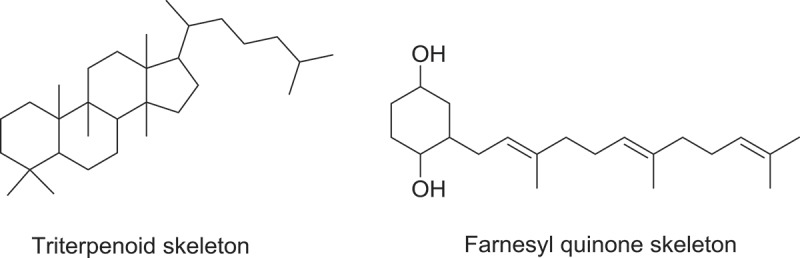
Parent carbon skeletons of triterpenoid and farnesyl quinone type of polyketide from *Ganoderma* sp. with antimicrobial and anti-parasitic activities.

Chemical analysis of numerous *Ganoderma* sp. has showed Ganoderma Triterpenes (GTs) are mainly lanostanoid-type triterpene (Zhang et al. 2015). Among them, majority contain 30 or 27 carbon atoms, and some occasionally may contain 24 carbon atoms. These compounds possess the same parent skeleton, namely a *trans-*configuration of rings A/B, B/C, C/D and 10*β*, 13*β*, 14*α*, 17*β* substituent. In addition, the substituents are always found at the C-3, 7, 11, 12, 15, 22, 23, 24 and 25 positions of the parent nucleus (Xia et al. 2014). Thirty carbon terpenoids are usually formed by the fusion of two smaller terpenoids precursors, each containing 15 carbons sesquiterpene. Head-to-tail fashions linking of isoprene units to form linear chains and various cyclisations and rearrangements is the core mechanism to give cyclic terpenoids (Mothana et al. 2000; Hill & Connolly 2013). The parent carbon skeleton of antimicrobial and anti-parasitic GTs is shown in Figure 1, from which it can be concluded that GTs are the most common antimicrobial and anti-parasitic compounds reported from *Ganoderma* sp.

Farnesyl quinone, a polyketide type, is the second most common antimicrobial and anti-parasitic compound from *Ganoderma* sp. Quinones are known to be oxidised derivatives of aromatic compounds and are often readily made from reactive aromatic compounds with electron-donating substituent such as catechols and phenols. Besides GTs, polypeptides, small peptides such as ganodermin, polysaccharide such as sacchachitin, and chitosan also possess antimicrobial and anti-parasitic properties (Mothana et al. 2000; Wang & Ng 2006; Sanodiya et al. 2009; Chuang et al. 2013). Structures of antimicrobial and anti-parasitic compounds from *Ganoderma* sp. are shown in Figure 2.

**Figure 2.**
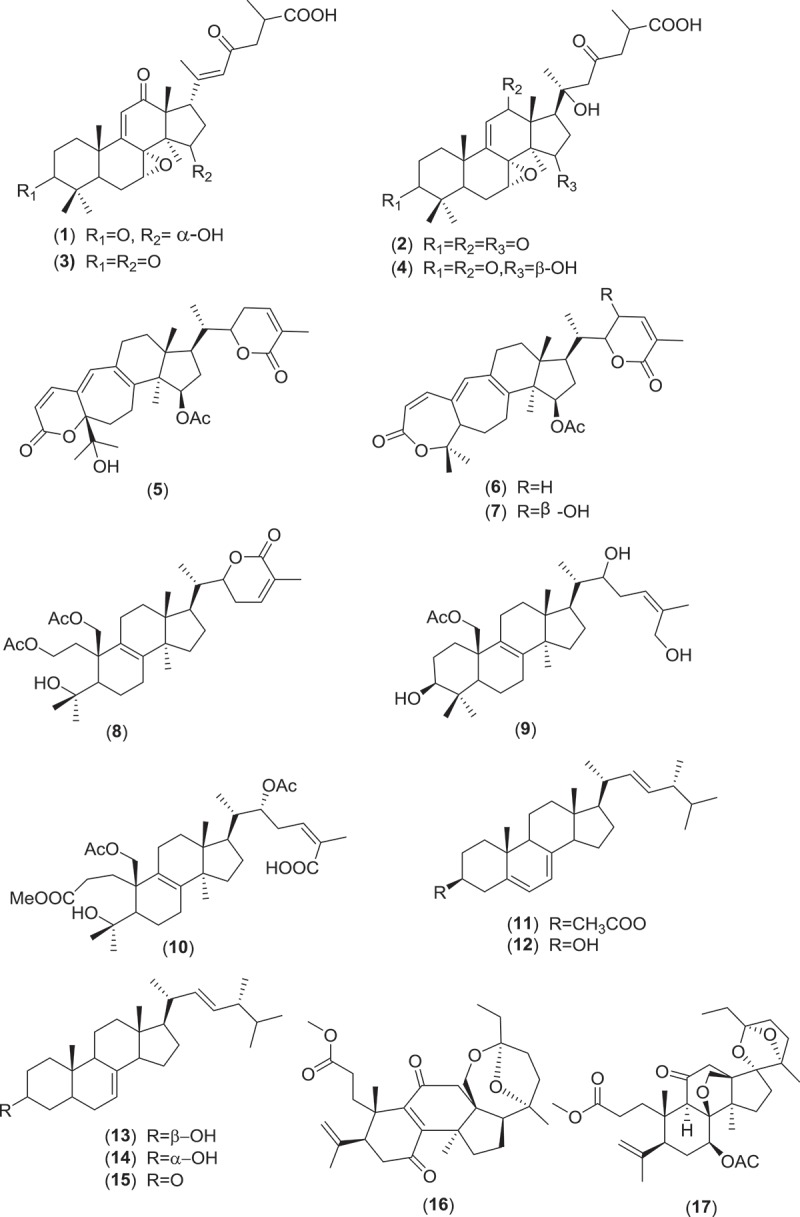
Structure of compounds with antimicrobial and anti-parasitic activities from *Ganoderma* sp.

**Figure 2.**
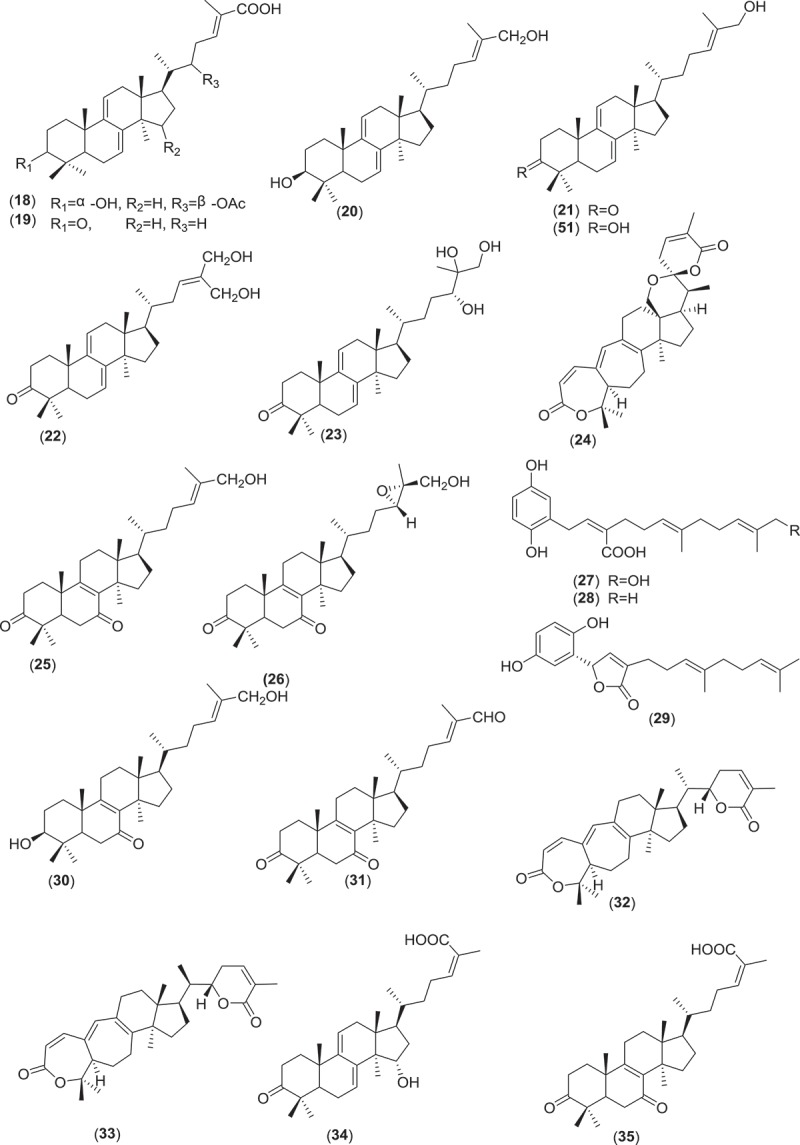
(Continued).

**Figure 2.**
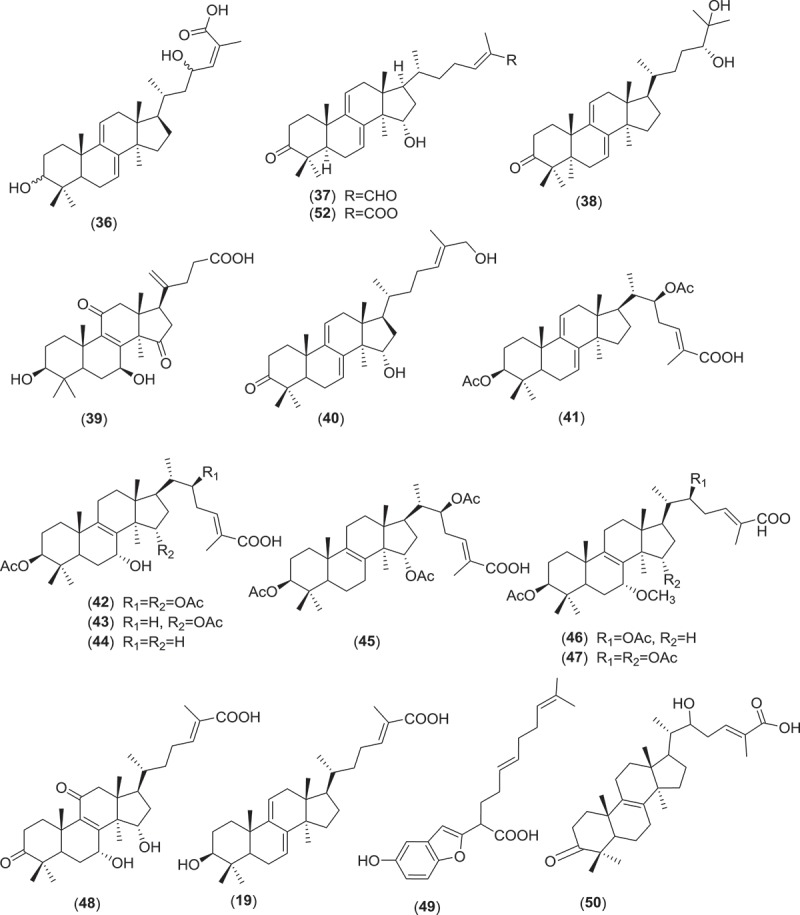
(Continued).

**Figure 2.**
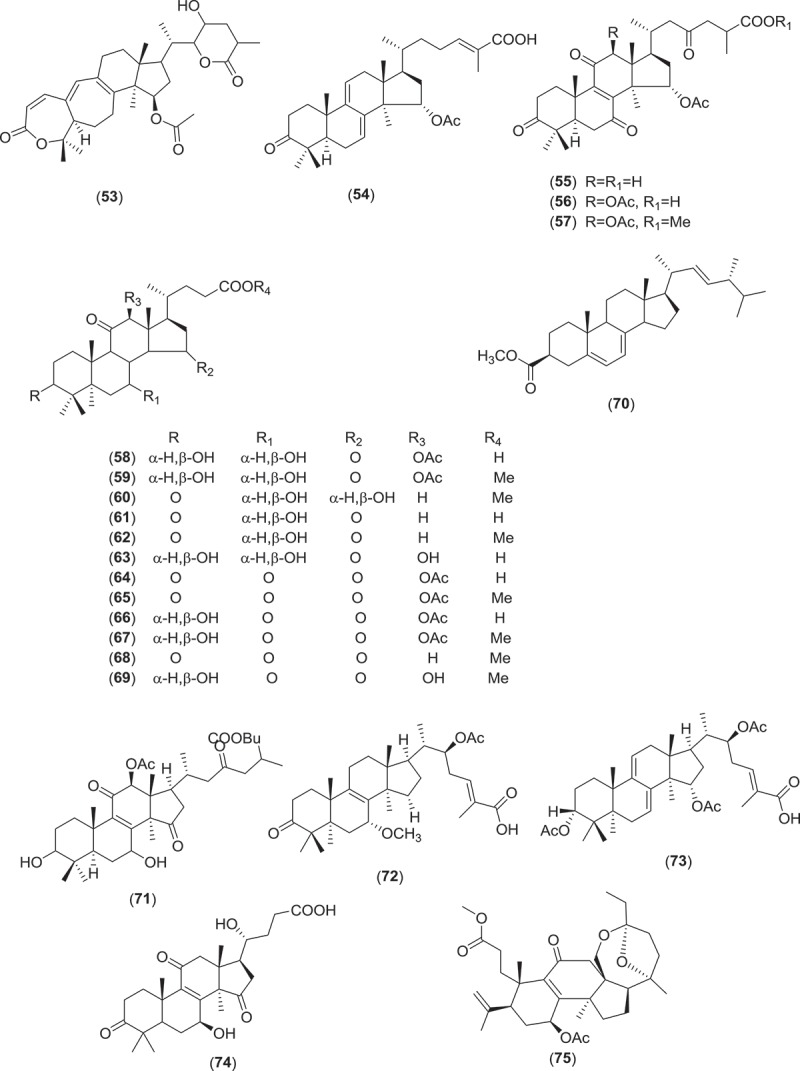
(Continued).

## 3. Isolation of antimicrobial and anti-parasitic bioactive compounds

**Figure 3.**
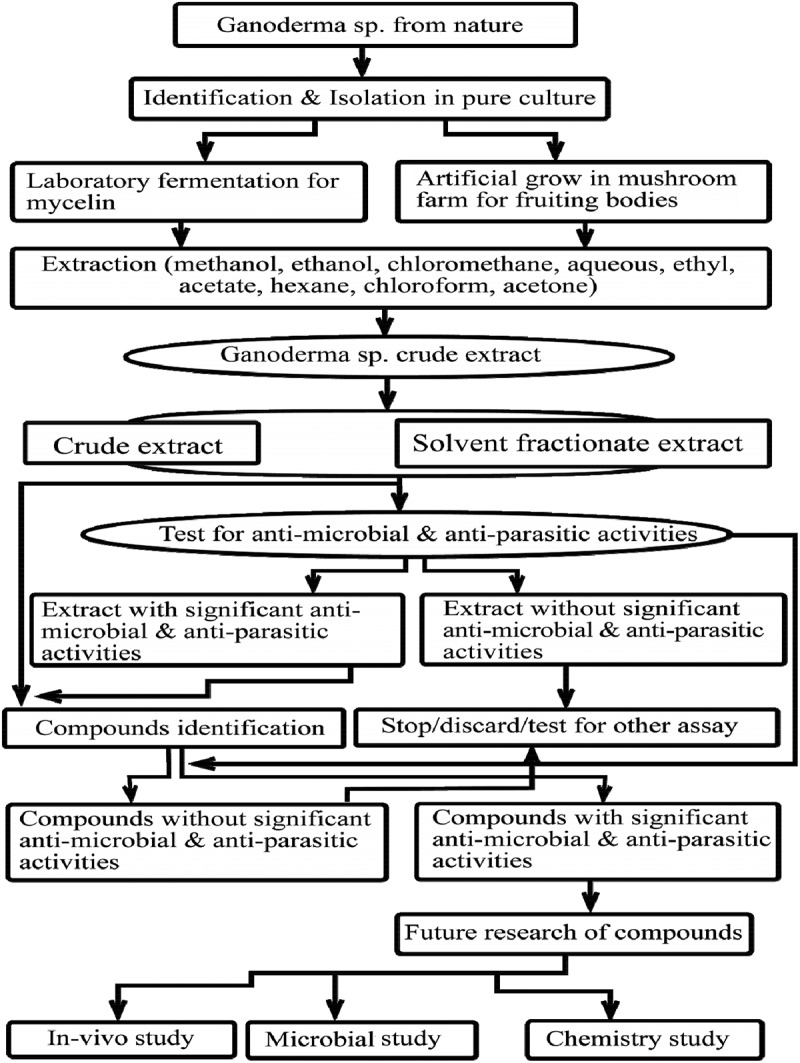
Flowchart of isolation of antimicrobial and anti-parasitic compounds from *Ganoderma* sp.

Extracts from fruiting bodies, both wild and cultivated, and mycelia from fermentation broth (Tables 1–4) are used for the isolation of antimicrobial and anti-parasitic bioactive compounds. Literatures divulge that most commonly ethanol (EtoAc) (Tables 1–4) is used to prepare crude extract; sometimes some researchers preferred other solvents such as chloroform (CHCl_3_), EtOH, and acetone (Isaka et al. 2016). In addition, our review reveals that hexane and ether are poorly used for the preparation of extract from *Ganoderma* sp. Moreover, some techniques such as microwave, ultrasound and enzyme treatments can facilitate the breakdown of the cell wall (Ferreira et al. 2015). Solvents like MeOH, EtOH, CH_2_Cl_2_, CHCl_3_ and aqueous – both cold and hot – are used for further purifications and isolation. Techniques such as thin-layer chromatography (TLC), high-performance liquid chromatography (HPLC), and column chromatography (CC) are used to facilitate the purification and isolation process (Huie & Di 2004). The general procedures of the isolation of antimicrobial and anti-parasitic compounds are shown in Figure 3. In addition, this outline can be used for other chemical investigations from *Ganoderma* sp.

**Table 1.** Details of antibacterial activities of *Ganoderma* sp. parts, products and compounds.

*Ganoderma*sp.	Extraction Solvent	Parts/products/compounds	Tested bacteria strains	Method	MIC/MBC	References
*G. atrum*	EtOH soluble acidic components	Fruiting bodies	*S. aureus sub species Aureus, E. coli, B.subtilis, P. vulgaris*	Micro dilution	1.56-25mg/ml/3.125-25mg/ml	(Li et al. 2012)
*G. lucidium*	96%EtOH	Fruiting bodies	*H. pylori* ATCC 43504, *S. aureus* ATCC 26003	Micro plate Agar, Disc fusion Assay	<1.0mg/ml &<10mg/ml resp./ND	(Shang et al. 2013)
*G. colossum*	Hexane: CH_2_Cl_2_(2:7)	Colossolactone E(**6**), 23 hydroxycolossolactone E(**53**)	*B. subtilis*IMI 347329, *P.syringae var* IMI 34748(ACTCC 19310)	TLC Agar Overlay	ND	(Lauretta Nwanneka Ofodile et al. 2011)
*G. pfeifferiBres.*	CH_2_ Cl_2_	Ganomycins A- B(**27–28**)	*S. aureus*(ATCC 6538), *B. subtilis*(SBUG 14), *E. coli*(SBUG 13), *P. mirabilis* (SBUG 47), *S. marcescens*(SBUG 9), *M. flavus*(SBUG 16)	Micro dilution	2.5-25*µ*g/mL/ND	(Mothana et al. 2000)
*G. applanatum*	96%EtOH	Mycelia extract	*B. cereus* (clinical isolate), *M. flavus*ATCC10240, *S. aureus*ATCC6538, *L. monocytogenes*NCTC7973, *E. coli*ATCC35218, *E. cloacae* (human isolate), *P. aeruginosa*ATCC27853 *and S. typhimurium*ATCC13311	Colorimetric Microbial Viability Assay	1.16–1.90mg/ml/2.54–4.00mg/ml	(Cilerdzic et al. 2016)
*G. carnosum*	96%EtOH	Mycelia extract	*B. cereus* (clinical isolate), *M. flavus*ATCC10240, *S. aureus*ATCC6538, *L. monocytogenes*NCTC7973, *E. coli* ATCC35218, *E. cloacae* (human isolate), *P. aeruginosa*ATCC27853 *and S. typhimurium*ATCC13311	Colorimetric Microbial Viability Assay	1.16–4.00mg/ml/1.16–4.00mg/ml	(Cilerdzic et al. 2016)
*G. lucidium*	96%EtOH	Mycelia extract	*B. cereus* (clinical isolate), *M. flavus*ATCC10240, *S. aureus*ATCC6538, *L. monocytogenes*NCTC7973, *E. coli* ATCC35218, *E. cloacae* (human isolate), *P. aeruginosa*ATCC27853 *and S. typhimurium*ATCC13311	Colorimetric Microbial Viability Assay	1.00–1.67mg/ml/1.16-400mg/ml	(Cilerdzic et al. 2016)
*G. colossusm*	CH_2_Cl_2_, MeOH, H_2_O	Fruiting bodies	*S. aureus*(ATCC 29213), *B. subtilis*(ATCC 6059), *E. coli* (ATCC 25922),*P. aeruginosa*(ATCC 27853), *M. flavus*(SBUG 16)	Agar Diffusion	ND	(Al-Fatimi et al. 2005)
*G. resinaceum*	CH_2_Cl_2_, MeOH, H_2_O	Fruiting bodies	*S. aureus (ATCC 29213), B. subtilis*(ATCC 6059), *E. coli* (ATCC 25922),*P. aeruginosa*(ATCC 27853), *M. flavus*(SBUG 16)	Agar Diffusion	ND	(Al-Fatimi et al. 2005)
*G. applanatum*	MeOH	Fruiting bodies	*E.coli*(ATCC 25922)	Micro dilution	ND	(Zengin et al. 2015)
*G.lucidum*	EtOH and H_2_O	Fruiting bodies	*S. aureus*(MTCC 96), *B. cereus* (MTCC 430), *P. aeruginosa*(MTCC 424)	Micro dilution	80-200mg/ml/ND	(Karwa & Rai 2012)
*G. lucidum*	Hexane and chloroform	Fruiting bodies	*S. aureus*(ATCC 6538), *B. subtilis*(ATCC 6633)	Agar Diffusion	6.25mg/ml/ND	(Vazirian et al. 2014)
*G. lucidum*	Hexane and chloroform	Ergosta-5,7,22-trien-3β-yl acetate(**11**),ergosta-7,22-dien-3*β*-yl acetate(**70**),ergosta-7,22-dien-3-one(**15**), ergosta-7,22-dien-3β-ol(**13**), ergosta-5,7,22 trien,-3β-ol(**12**), ganodermadiol(**20**)	*S. aureus*(ATCC 6538), *B. subtilis*(ATCC 6633)		2.5-5mg/ml/ND	(Vazirian et al. 2014)
*G.lucidum*	Hot H_2_O	Carpophores	*B. anthracis*ATCC 6603, *B. cereus* ATCC 27348, *B. subtilis*ATCC 6633, *M. luteus* ATCC 9341, *S. aureus* ATCC 25923,*E.coil*ATCC 259 22, *K. oxytoca*ATCC 8724, *K. pneumoniae*ATCC 10031, *P. vulgaris* ATCC 27853, *S. typhi*ATCC 6229	Micro dilution	1.25–5.0mg/ml/ND	(Yoon et al. 1994)
*G. lucidium*	96% EtOH	Basidiocarps	*B. cereus* (clinical isolate), *M. flavus*ATCC10240, *S. aureus*ATCC6538, *L. monocytogenes*NCTC7973, *E. coli* ATCC35218, *E. cloacae* (human isolate), *P. aeruginosa*ATCC27853 & *S. typhimurium*ATCC13311	Disc-diffusion & Micro dilution	1–3.4mg/ml/1.4–4.0mg/ml	(Ćilerdžić et al. 2014)
*G.lucidium*	95% EtOH	12b-acetoxy-3*β*,7 *β* -dihydroxy-11,15,23-trioxolanost-8-en-26-oic acid butyl ester(**71**)	*S. aureus*(ATCC 6538) & *B.subtilis*(ATCC6633)	Micro dilution	68.5 *µ*M&123.8 *µ*M	(Liu et al. 2014)
*G. lucidum*	MeOH	NG	*S. aureus*(ATCC 6538),*B.cereus*(clinical isolate), *L. monocytogenes*(NCTC 7973), *M. flavus*(ATCC 10240), *P. aeruginosa*(ATCC 27853), *E. coli* (ATCC 35210), *S. typhimurium*(ATCC 13311), *E. cloacae* (human isolate)	Micro dilution	0.0125–0.75mg/ml/0.035–1.5mg/ml	(Heleno et al. 2013)
*G. lucidium*	H_2_O	Mycelia(Protein extract)	*S. epidermidis, B. subtilis, B .cereus E. coli, P. aeruginosa*	Micro dilution	20–81.5mg/ml/ND	(Sa-Ard et al. 2015)
*G. lucidium*	H_2_O	Fruiting bodies(Protein extract)	*S. epidermidis, S. aureus, B. subtilis, B. cereus, E. coli, P. aeruginosa*		81.5-512mg/ml/ND	(Sa-Ard et al. 2015)
*G. orbiforme*	MeOH, EtOAc, Acetone	Mycelia#	*M. tuberculosis*	Green Fluorescent Protein Micro Plate Assay	0.781-50*µ*g/mL/ND	(Isaka et al. 2016)

MeOH-Methanol; EtOH-Ethanol; dH_2_O- Distilled water; NG- Data Not Given; ZOI-Zone Of Inhibition; MIC- Minimum Inhibitory Concentration; MBC- Minimum Bactericidal Concentration; *S. aureus- Staphylococcus aureus; B. subtilis- Bacillus subtilis*;, #(astraodoric acid B(**50**), ganorbiformin F(**72**), ganoderic acid TR(**34**), ganoderic acid T(**73**), ganoderic acid S(**18**), (22*S*,24*E*)-3*β*,15*α*,22-triacetoxylanosta-8,24-dien-26-oic acid(**41**), (24*E*)-3*β*-acetoxy-7*α*-hydroxylanosta-8,24-dien-26-oic acid(**44**), (24*E*)-3*β*,15*α*-diacetoxy-7*α*-hydroxylanosta-8,24-dien-26-oic acid(**43**), (22*S*,24*E*)-7*α*-hydroxy-3*β*,15*α*,22-triacetoxylanosta-8,24-dien-26-oic acid(**42**), (22*S*,24*E*)-3*β*,22-diacetoxy-7*α*-methoxylanosta-8,24-dien-26-oic acid(**46**), (22*S*,24*E*)-7*α*-Methoxy-3*β*,15*α*,22-triacetoxylanosta-8,24-dien-26-oic acid(**47**), (22*S*,24*E*)-3β,22-diacetoxylanosta-7,9(11),24-trien-26-oic acid(**45**).

**Table 2.** Illustration of antifungal activities of *Ganoderma* sp. parts, products and compounds.

*Ganoderma*sp.	Extraction Solvent	Parts/products/compounds	Tested Fungal strains	Method	Antifungal Concentration/ZOI/MIC/MFC/EC_50_ value	References
*G. colossus*	MeOH	Fruiting bodies	*C. maltosa*	Agar Diffusion Assay	8mm/2mg/disc-ZOI	(Al-Fatimi et al. 2005)
*G*. *applanatum*	MeOH& H_2_O	NG	*C. albicans and C. parasilopsis*	Broth Micro dilution	1.25 & 2.5mg/ml-Antifungal activity	(Zengin et al. 2015)
*G*. *resinaceum*	MeOH& H_2_O	NG	*C. albicans and C. parasilopsis*	Broth Micro dilution	1.25 & 2.5mg/ml-Antifungal activity	(Zengin et al. 2015)
*G*. *lucidium*	96%EtOH	Fruiting bodies	*Acremonium strictum*BEOFB10m, *A. glaucus*BEOFB21m, *A. flavus*BEOFB22m, *A.fumigatus*BEOFB23m, *A.nidulans*BEOFB24m, *A.niger*BEOFB25m, *A. terreus*BEOFB26m, *T. viride*BEOFB61m	Disc-diffusion & Micro dilution	0.5-308mg/ml-MIC; 1.0–4.0mg/ml-MFC	(Ćilerdžić et al. 2014)
*G.lucidium*	MeOH	Fruiting bodies	*A. fumigatus*(human isolate), *A. versicolor*(ATCC 11730), *A. zochraceus*(ATCC 12066), *A. niger*(ATCC 6275), *T. viride*(IAMz5061), *P. funiculosum*(ATCC 36839), *P. ochrochloron*(ATCC 9112)*and P. verrucosum var*. cyclopium (food isolate)	Micro dilution	0.005–1.5mg/ml-MIC; 0.1–4.5mg/ml-MFC	(Heleno et al. 2013)
*G.lucidium*	EtOH& chemical synthesis	RE–CGAP(RE: La, EuandYb)	*V. mali, F. oxysporum, G. graminis, C. gloeosporioides, A. brassicae*	Disc diffusion	1.85–568.30mg/ml-EC_50_	(Sun et al. 2014)
*G.lucidium*	dH_2_O	Ganodermin	*Botrytis cinerea, F. oxysporum and Physalo sporapiricola*	Paper Disks	8.1–12.4mM-Antifungal	(Wang & Ng 2006)
*G.annulare*	NG	Applanoxidic acids A(**1**), C(**2**) & F(**3**)	*M. cannis & T. mentagrophytes*	Micro dilution	500 to 1000mg/ml-Antifungal	(Smania et al. 2003)
*G*. *applanatum*	96%EtOH	Mycelia	*Acremonium strictum, A. glaucus, A. flavus, A. fumigatus, A. nidulans, A. niger, A. terreus, T. viride*	Colorimetric	1.00–2.00mg/ml-MIC; 1.17–4.00mg/ml-MFC	(Cilerdzic et al. 2016)
*G*. *carnosum*	96%EtOH	Mycelia	*Acremonium strictum, A. glaucus, A. flavus, A. fumigatus, A. nidulans, A. niger, A. terreus, T. viride*	Colorimetric	0.83–2.00mg/ml- MIC; 2.00–3.33mg/ml-MFC	(Cilerdzic et al. 2016)
*G*. *lucidum*	96%EtOH	Mycelia	*Acremonium strictum, A. glaucus, A. flavus, A. fumigatus, A. nidulans, A. niger, A. terreus, T.viride*	Colorimetric	0.50–2.00mg/ml- MIC; 1.17–4.00mg/ml-MFC	(Cilerdzic et al. 2016)

MeOH: Methanol; EtOH: Ethanol; dH_2_O: Distilled water; NG: Data Not Given; ZOI: Zone Of Inhibition; MIC: Minimum Inhibitory Concentration; MFC: Minimum Fungicidal Concentration; EC_50_: Concentration; RE–CGAP: Rare Earth-CarboxymethylatedGanodermaapplanatum Polysaccharide; *μ*M: Micro Mole; mg/ml: milligram/millilitre.

**Table 3.** Illustration of antiviral activities of *Ganoderma* sp. parts, products and compounds.

*Ganoderma*sp.	Tested Viral strains	Extraction Solvent	Parts/products/compounds	Method	IC_50_ (≤50*µ*M)/EC_50_/ED_50_ value	References
*G. sinense*	HIV 1(HIV-1 protease)	CHCl_3_	Ganoderic acid GS-2(**48**), 20-hydroxylucidenic acid N(**74**), 20(21)-dehydrolucidenic acid N(**39**) & ganoderiol F(**22**)	*In vitro* (Enzymatic)	20 – 40*µ*M	(Sato et al. 2009)
*G. colossum*	HIV 1(HIV-1 protease)	CHCl_3_	Colossolactone V(**10**), Colossolactone VII(**8**), Colossolactone VIII(**7**), Schisanlactone A(**33**), Colossolactone G(**5**), Colossolactone A(**9**)	*In vitro* (Enzymatic)	5-39*μ*g/mL	(El Dine et al. 2008)
*G. colossum*	HIV 1(HIV-1 protease)	CHCl_3_	Ganomycin I(**29**) &Ganomycin B(**28**)	*In vitro* (Enzymatic)	7.5 and 1.0 *μ*g/mL	(El Dine et al. 2009)
*G.lucidium*	HIV 1(HIV-1 protease)	MeOH	Ganoderiol F(**21**) &Ganodermanontriol(**23**)	*In vitro* (Enzymatic)	7.8*μ*g/mL	(El-Mekkawy et al. 1998)
*G. lucidum*	Herpes Simplex Virus types 1 (HSV-1) and 2 (HSV-2), Influenza A virus (Flu A) and Vesicular Stomatitis Virus (VSV) Indiana and New Jersey strains	H_2_O &MeOH	Carpophores	Cytopathic Effect (CPE) Inhibition Assay & Plaque Reduction Assay	68-1790*μ*g/mL-EC50	(Eo et al. 2000)
*G. lucidum*	HSV-1 and HSV-2	H_2_O/EtOH	Acidic protein bound polysaccharide	Plaque Reduction Assay		(Eo et al. 2000)
*G. lucidum*	Oral Human Papillomavirus (HPV)	NG	Fruiting bodies	*In vivo* (Human)	87% clearance of virus	(Donatini 2014)
*G. lucidum*	Newcastle Disease Virus(anti-neuraminidase)	MeOH, EtOAc & Butanol		*In vitro*	Virus dilution ratio(1:16, 1:16, 1:32)	(Shamaki et al. 2014)
*G. lucidum*	Epstein-Barr Virus	MeOH	Fruiting bodies*	*In vitro*	96–100% at 1 103 mol ratio/TPA	(Iwatsuki et al. 2003)
*G. lucidum*	Hepatitis B virus	NG	mycelia	*In vitro* (HepG2 cells)	IRA(HBsAg, HBeAg) up to 100%	(Y. Li et al. 2006)
*G. lucidum*	Hepatitis B	H_2_Oand CHCl_3_	mycelia(Ganoderic acid)	*In vitro* (HepG2215)	Inhibition of production of HBV surface antigen and HBVe at 8*μ*g/mL	(Y.-Q. Li & Wang 2006)
*G. pfeifferi*	Influenza virus type A and HSV type 1	NG	Ganodermadiol(**20**), lucidadiol (**30**) & applanoxidic acid G(**4**)	Dye Uptake Assay	Influenza ED50(0.19–0.22mmol/l); HSV 1(0.068 mmol/l for ganodermadiol)	(MothanaRa et al. 2003)
*G. pfeifferi*	HSV type 1	CH_2_Cl_2_	Ganoderone A(**25**), Lucialdehyde B(**31**), Ergosta-7,22-dien-3α-ol(**14**), Ganoderol A(**21**) & Ganoderol B(**51**)	*In vitro* (Vero cells)	0.03–0.75*μ*g/mL(IC50)	(Niedermeyer et al. 2005)
*G. pfeifferi*	Influenza virus type A	CH_2_Cl_2_	Ganoderone C(**26**), Lucialdehyde B(**31**) & Ergosta-7,22-dien-3α-ol(**14**)	*In vitro* (MDCK cells))	0.78–2.6*μ*g/mL(IC50)	(Niedermeyer et al. 2005)
*G. lucidum*	Enterovirus 71	NG	Lanosta-7,9(11),24-trien-3- one,15;26-dihydroxy (GLTA)(**40**), Ganoderic acid Y(**19**)	*In vitro* (Human Rhabdomyosarcoma)	0.16 to 4 *μ*g/ml(IC50)	(W. Zhang et al. 2014)

MeOH: Methanol; EtOH Ethanol; H_2_O: water; NG: Data Not Given; IC_50_: half-maximal Inhibitory Concentration; EC_50_: half-maximal Effective Concentration; ED_50_: median effective dose; *μ*M: Micro Mole; mg/m: -Milligram/Millilitre; *μ*g/ml: Microgram/millilitre; *(Lucidenic acid P(**58**), Methyl lucidenate P(**59**), Methyl lucidenate Q(**60**), Lucidenic acid A(**61**), Methyl lucidenate A(**62**), Lucidenic acid C(**63**), Lucidenic acid D2(**64**), Methyl lucidenate D2(**65**), Lucidenic acid E2(**66**), Methyl lucidenate E2(**67**), Methyl lucidenate F(**68**), Methyl lucidenate L(**69**), Ganoderic acid E(**54**), Ganoderic acid F(**57**), Methyl ganoderate F(**56**), Ganoderic acid T-Q(**54**)).

**Table 4.** Details of anti-parasitic activities of *Ganoderma* sp. parts and compounds.

*Ganoderma*sp.	Extraction Solvent	Parts/compounds	Test Parasite	Method	LD_50_/IC_50_ value	References
***Ganoderma*sp.**	EtOAc&MeOH	Fruiting bodies(schisanlactone B(**32**), Ganodermalactone F(**24**), colossolactone E(**6**))	*P. falciparum*	Micro culture Radioisotope Technique	6.0–10.0 *μ*M	(Lakornwong et al. 2014)
***G. lucidum***	EtOAc&MeOH	Fruiting bodies*	*P. falciparum*	Micro culture Radioisotope Technique	6.0-20*μ*M	(Adams et al. 2010)
***G. boninense***	EtOH	Ganoboninketals A(**75)** Ganoboninketals B-C(**16–17**)	*P. falciparum*	DNA Fluorescence Signal Test	4.0, 7.9, and 1.7 *μ*M	(Ma et al. 2014)
***G. lucidum***	EtOH	Crude extract	*P. berghei*	*In Vivo* Malarial activity		(Oluba et al. 2012)
***G.lucidum***	NG	Lectin	*H. glycines*	Parasite Mortality Test	(>10 mg/ml/2hrs,4.5 mg/ml/24hrs, 1.7 mg/ml/48hrs	(Zhao et al. 2009)
***G. lucidum***	NG	Lectin	*D. dipsaci*	Parasite Mortality Test	>10 mg/ml	(Zhao et al. 2009)

EtoAc: Ethyl Acetate; EtOH: ethanol; MeOH: Methanol; *P. falciparum*: *Plasmodium falciparum; H. glycines*: *Heteroderaglycines; D. dipsaci*: *Ditylenchusdipsaci*; NG: Data Not Given; *μ*M: Micro Mole; mg/ml: milligram/millilitre; *(Ganodericacid DM(**35**), Ganoderic Acid TR 1(**52**),Ganoderic Aldehyde TR(**37**),23-Hydroxyganoderic Acid S(**36**), Ganoderic acid S(**18**), Ganodermanondiol(**37**), Ganofuran B(**49**)).

## 4. Antibacterial activities of compounds and extracts of *Ganoderma* sp

Currently bioassay-guided antibiotics identification, TLC and chromatography bio-autography are used to track antibacterial ingredients from the extract (Huie & Di 2004). Minimum inhibitory concentration (MIC) and 50% inhibitory concentration (IC_50_) values are used to determine the potency of antibacterial agents. Our literatures review showed that MeOH and EtOH are good solvents for the extraction of antibacterial compounds of interest rather than other organic solvents; however, the parts of *Ganoderma* sp. used and the tested bacterial strains may be the limiting factors in choosing the solvent. Most studies that use alcoholic solvents for extraction showed very low MIC (Li et al. 2012; Shang et al. 2013; Cilerdzic et al. 2016). Several studies on the fruiting bodies of *Ganoderma* sp. reveal that the compounds have the inhibitory ability to the different types of Gram positive bacteria (GPB), Gram negative bacteria (GNB) including the mycobacteria (Al-Fatimi et al. 2005; Isaka et al. 2016).

Colossolactone E (**6**) and 23-hydroxycolossolactone E (**53**), two colossolactones-triterpenes, were active against *Bacillus subtilis* and *Pseudomonas syringae*. However, the researcher did not determine the MIC and MBC of compounds against this bacterium (Ofodile et al. 2011). Moreover, two hydroquinones, ganomycins A (**27**) and B (**28**), were found to be the most effective to inhibit the bacterium. The MIC values of compounds **27** and **28** were 25 *µ*g/ml against *Staphylococcus aureus* and 2.5 *µ*g/ml against *Micrococcus flavus,* respectively, taking positive control ampicillin (MIC = 0.05 *µ*g/ml and 0.25 *µ*g/ml for *S. aureus* and *M. flavus,* respectively). In addition, in agar diffusion assay Zone of Inhibition (ZOI) 15–25 mm/100 *µ*g/paper disk was found for GPB such as *B. subtilis, S. aureus* and *M. flavus*. However, *P. aeruginosa, Candida albicans* and *C. maltose* at 100 *µ*g/paper disk did not respond to these compounds (Mothana et al. 2000). In a work performed by Isaka et al. (2016), EtOAc and MeOH extract of *Ganoderma* sp. BCC 16,642 isolated different compounds astraodoric acid C (**50**), ganorbiformin F (**72**), ganoderic acid TR (**34**), ganoderic acid T (**73**), ganoderic acid S (**18**), lanostanoid, ((22*S*,24*E*)-3*β*,15*α*,22-triacetoxylanosta-8,24-dien-26-oic acid (**45**), (24*E*)-3*β*-acetoxy-7*α*-hydroxylanosta-8,24-dien-26-oic acid (**44**), (24*E*)-3*β*,15*α*-diacetoxy-7*α*-hydroxylanosta-8,24-dien-26-oic acid (**43**), (22*S*,24*E*)-7*α*-hydroxy-3*β*,15*α*,22-triacetoxylanosta-8,24-dien-26-oic acid (**42**), (22*S*,24*E*)-3*β*,22-diacetoxy-7*α*-methoxylanosta-8,24-dien-26-oic acid (**46**), (22*S*,24*E*)-7*α*-methoxy-3*β*,15*α*,22-triacetoxylanosta-8,24-dien-26-oic acid (**47**), (22*S*,24*E*)-3*β*,22-diacetoxylanosta-7,9(11),24-trien-26-oic acid (**41**), which were observed to be active against the *Tubercular bacilli* with the MIC value in the range of 0.781–50 *µ*g/ml. In another study, steroidal compounds like ergosta-5,7,22-trien-3*β*-yl acetate (**11**), ergosta-5,7,22-dien-3*β*-yl acetate (**70**), ergosta-7,22-dien-3-one (**15**), ergosta-7,22-dien-3*β*-ol (**13**), ergosta-5,7,22-trien-3*β*-ol (**12**) and ganodermadiol (**20**) were found to be effective against *S. aureus* and *B. subtilis* with MIC value of 2.5–5 mg/ml (Vazirian et al. 2014). Ethanolic and EtOAc extract compounds 12*β*-acetoxy-3*β*, 7*β*-dihydroxy-11, 15, 23-trioxolanost-8-en-26-oic acid butyl ester (**71**) from fruiting bodies of *G. lucidium* showed significant inhibition against *S. aureus* and *B. subtilis* with MIC values of 68.5 *µ*M and 123.8 *µ*M, respectively (positive control ampicillin = 4.1 *µ*M and 19.3 *µ*M, resp.) (Liu et al. 2014).

Literatures reveal most of the antibacterial tests are performed on crude extract with significant effective results rather than pure compounds (Sa-Ard et al. 2015; Zengin et al. 2015; Cilerdzic et al. 2016). In addition, scanty information is available on the in vivo model test of effective compounds; we noticed only compounds (**27**) and **28** have been tested the in vivo model of the Methicillin-resistant Staphylococcus aureus (MRSA)-infected mouse (Mikolasch et al. 2016).

## 5. Antifungal activities of compounds and extracts of *Ganoderma* sp

An antifungal protein – ganodermin – isolated from the fruiting bodies of *G. lucidium* inhibits the growth of *Botrytis cinerea, Fusarium oxysporum* and *Physalo sporapiricola* with an IC_50_ value of 15.2 mM, 12.4 mM and 18.1 mM, respectively (Wang & Ng 2006). Terpeneoids like applanoxidic acids A (**1**), C (**2**) and F (**3**) isolated from *G. annulare* inhibit the growth of the fungi *Microsporum cannis* and *Trichophyton mentagrophytes* at concentrations of 500–1000 *µ*g/ml (Smania et al. 2003).

In another study, researchers synthesised the complexes of polysaccharide with different rare earth metal (RE–CGAP (RE: La, Eu and Yb)) and evaluated their efficacy against fungi and reported that rare earth carboxymethylated *G. Applanatum* polysaccharide (RE-CGAP) complexes with antifungal activities with EC_50_ value of 1.01–28.48 mg/ml (>100 mg/ml not included) (Sun et al. 2014). The details of the antifungal action of *Ganoderma* sp. are demonstrated in Table 2.

## 6. Antiviral activities of compounds and extracts from *Ganoderma* sp

It is interesting to note that the majority of antiviral investigations on *Ganoderma* sp. have been performed from fruiting body against the protease enzyme of HIV virus. The compounds ganoderiol F (**22**) and ganodermanontriol (**23**) were found to be active as anti-HIV-1 agents with an inhibitory concentration of 7.8 *µ*g/ml. In addition, in the same experiment ganoderiol B (**51**), ganoderiol A (**21**), ganoderic acid A (**76**), ganoderic acid B (**77**), ganoderic acid C1 (**78**) and ganoderic acid H (**79**) were found to be moderate in their efficacy (El-Mekkawy et al. 1998; El Dine et al. 2008). Colossolactone types of triterpenoids such as colossolactone V (**10**), colossolactone VII (**8**), colossolactone VIII (**7**), schisanlactone A (**33**), colossolactone G (**5**) and colossolactone A (**9**) were isolated from the chloroform extract from *G. lucidium* with and IC_50_ value of 5–39 *μ*g/ml (El Dine et al. 2008). Similarly in Sato et al. (2009), isolated lanostane-type triterpenoids-ganoderiol F (**22**), ganoderic acid GS-2 (**48**) and 20-hydroxylucidenic acid N (**74**), 20(21)-dehydrolucidenic acid N (**39**) from CHCl_3_ extract of the fruiting body of *G. sinense* and demonstrated the anti-HIV-1 protease activity with IC_50_ values of 20–40 *μ*M (El Dine et al. 2008; Sato et al. 2009). Compounds from the CHCl_3_ extract of the fruiting bodies of *G. colossum*, farnesyl hydroquinone, ganomycin I (**29**) and ganomycin B (**28**), competitively inhibit the active site of HIV-1 protease enzyme with IC_50_ values of 7.5 and 1.0 *μ*g/ml, respectively (El Dine et al. 2008).

*Ganoderma pfeifferi* triterpenes, ganodermadiol (**20**), lucidadiol (**30**) and applanoxidic acid G (**4**), were active against influenza virus type A with ED_50_ of greater than 0.22 mM, 0.22 mM and 0.19 mM, respectively (MothanaRa et al. 2003). Similarly others triterpenes such as ganoderone C (**26**) (IC_50_: 2.6 *µ*g/ml), lucialdehyde B (**31**) (IC_50_:3.0 *µ*g/ml) and ergosta-7, 22-dien-3*α*-ol (**14**) (IC_50_: 0.78 *µ*g/ml) inhibited the growth of Madin-Darby canine kidney (MDCK) cells infected with influenza virus (Niedermeyer et al. 2005). Herpes simplex virus were inhibited by triterpenes such as compound (**20**) (ED_50_: 0.068 mM), ganoderone A (**25**) (IC_50_:0.075 *µ*g/ml), (**31**) (IC_50_:0.03 *µ*g/ml) and compound **14** (IC_50_: 0.03 *µ*g/ml), whereas compounds **21** and **51** were less effective in comparison (MothanaRa et al. 2003; Niedermeyer et al. 2005). *G. lucidium* triterpenes lanosta-7, 9 (11), 24-trien-3-one, 15; 26-dihydroxy (GLTA) (**40**) and ganoderic acid Y (**19**) possess inhibitory action towards enterovirus 71 with IC_50_ value of 0.16–4 *μ*g/ml (Zhang et al. 2015). The details of the antiviral activities of *Ganoderma* sp. have been illustrated in Table 3.

## 7. Anti-parasitic activities of compounds and extracts from *Ganoderma* sp

Nortriterpenes-ganoboninketals A-C (**15–17**) obtained from the biochemical analysis of the fruiting bodies of *G. boninense* were found to possess anti-parasitic activity against *P. falciparum* with IC_50_ values of 4.0, 7.9 and 1.7 *μ*M, respectively (Adams et al. 2010; Ma et al. 2014). Similarly three triterpenes – schisanlactone B (**32**), ganodermalactone F (**24**) and colossolactone E (**6**) – isolated with EtOAc and MeOH from *Ganoderma* sp. KM01 are active against *P. falciparum* in the range 6.0−10.0 *μ*M (Lakornwong et al. 2014a). In addition, *G. lucidium* terepenes – ganoderic acid DM (**35**), ganoderic acid TR1 (**52**), ganoderic aldehyde TR (**37**), ganoderic acid S (**18**), ganodermanondiol (**38**) and ganofuran B (**49**) – isolated from EtOAc inhibit *P. falciparum* with IC_50_ value of range 6.0–20 *μ*M (Adams et al. 2010). In a recent study, Zhao et al. found lectin to be active against the plant nematodes *Heterodera glycines* and *Ditylenchus dipsaci*, though their potency was not significant to be used practically (Zhao et al. 2009) .

## 8. Conclusion and future perspective

*Ganoderma* sp. has been used for treatment in various diseases over a long period (Paterson 2006). Our review clearly showed that compounds from *Ganoderma* sp., under the extensive in vivo and pharmacological research, can be used in various microorganisms and parasitic diseases. However, the in vivo experiment and pharmacological research of the identified compounds are very limited. Therefore, future work should be focused on in vivo and pharmacological assays of known compounds, especially Ganoderma terpenes that have antimicrobial and anti-parasitic properties. A better understanding of the antimicrobial and anti-parasitic compounds from *Ganoderma* sp. is crucial for identifying the potential side effects and trace out the new host target and molecular mechanisms, which will provide evidence to further clinical applications of these compounds.

Although extensive researches have been carried out on *Ganoderma* sp., most of the studies were concentrated on few species, *G. lucidum* for instance. Researchers must need to pay more attention to closely related species based on the phylogenic analysis though numerous challenges including genetic analysis, biosynthetic metabolism, separation, isolation and identification may be encountered. In addition, due to the rapid emergence of drug resistance in microorganisms and parasites, fewer options have been left for the treatment of diseases caused by microorganism and parasites. To fight back this problem, further research should be focused on this field for all the identified compounds and the unidentified compounds, which are on the way to be identified. Our review revealed numerous extracts of *Ganoderma* sp. exhibit the inhibition to microorganisms including parasites, indicating that *Ganoderma* sp. in particular still seem to possess opportunities for new drug lead compounds.

Scanty literatures are found on the assay of identified compounds for animal and plants pathogens including parasites, indicating that this area of research for the *Ganoderma* sp. compounds is overlooked. Also, our current experience on a literatures review of *Ganoderma* sp. compounds, more than 430 compounds identified (Baby et al. 2015; Rai et al. 2015), most of the compounds have not been performed on the antimicrobial and anti-parasite assay. Therefore, further studies need to be carried out in order to explore this concealed area.

No doubt, it is evident that *Ganoderma* sp. is going to serve as one of the potential sources of novel antibiotics and anti-parasitic drugs in the near future. To reach the apex and specificity of effective antimicrobial and anti-parasite activity, cooperative investigations need to be carried out in the areas of genomic, bioinformatics, chemistry and pharmacology. Moreover, strategies to evoke the sleeping gene clusters linked for the production of bioactive compounds and its regulation need to be adopted.
